# Maternal high‐fat diet in mice leads to innate airway hyperresponsiveness in the adult offspring

**DOI:** 10.14814/phy2.13082

**Published:** 2017-03-08

**Authors:** Kelvin D. MacDonald, Aurelia R. Moran, Ashley J. Scherman, Cindy T. McEvoy, Astrid S. Platteau

**Affiliations:** ^1^ Department of PediatricsOregon Health and Science UniversityPortlandOregon

**Keywords:** Airway responsiveness, maternal diet, offspring

## Abstract

Maternal obesity prior to and during pregnancy has been associated with an increased incidence of childhood asthma. As diets rich in saturated fat are linked to obesity and inflammation, we created a murine model to investigate the effect of maternal high‐fat diet (HFD) on adult offspring airway hyperreactivity (AHR), a cardinal feature of asthma. Balb/cByJ dams were fed a HFD (60% fat Calories) or normal‐fat diet (NFD) (10% fat Calories) from 8 weeks prior to first breeding through their pregnancies. Pups were weaned to either a HFD or NFD (at 4 weeks of age). AHR was measured in the 10‐week‐old offspring following inhaled methacholine challenge by end‐inflation technique. Bronchial alveolar lavage fluid (BALF) was analyzed for cell count, total protein, and IL‐6. Offspring of HFD dams weaned to NFD had increased AHR compared to offspring of NFD dams weaned to NFD. Offspring of HFD dams that remained on HFDs had increased AHR compared to offspring of NFD dams weaned to HFDs. Offspring of HFD dams had higher BALF cell counts, higher neutrophil percentage, greater total protein, and IL‐6 in the BALF. These results demonstrate that a maternal diet high in saturated fat through pregnancy and lactation plays a key role in programming adult offspring AHR.

## Introduction

Asthma rates have increased in the United States from 7.3% in 2001 to 8.4% in 2010 (Akinbami et al. 2012). By 6 years of age, 50% of children will have had respiratory symptoms of wheezing or cough that require medical attention, contributing to significant healthcare expenditures (Bush 2007). Increasing respiratory morbidity in children is likely multifactorial, one proposed explanation is the growing incidence of maternal obesity. Multiple studies demonstrate associations between maternal pregnancy body mass index >30 m/kg^2^ and increased incidence of childhood wheezing and asthma symptoms in the offspring. A sample of over 6k mother‐baby pairs clearly showed increased bronchodilator use in offspring of obese pregnancies (MacDonald et al. 2016). In a prospective study, Pike et al. (2013) reported associations between obese pregnancy and increased wheezing history in offspring thru age 5 years but no association with asthma or atopy. Fourteen separate studies relating maternal obesity and pregnancy weight gain to childhood asthma and wheeze have been summarized by Forno et al. (2014). Importantly, not all wheezing children go on to have asthma (Morgan et al. 2005; Bush 2007) and mounting evidence exists identifying distinct molecular and clinical asthma phenotypes (Wenzel 2012). Our model allows for controlled pre and postnatal high‐fat diet (HFD) exposures and analysis of offspring airway hyperreactivity (AHR) by bronchial provocation, an indicator of asthma, which has not been studied in humans.

High calorie diets, particularly those with excessive amounts of saturated fats, directly contribute to obesity (Bray and Popkin 1998). Diets high in saturated fats are linked to chronic systemic inflammation and metabolic dysregulation in humans and rodents. Maternal obesity has additional long‐term health risks for the offspring including cardiovascular disease and metabolic disorders which have been documented in human studies and animal models (Vasudevan et al. 2011; O'Reilly and Reynolds 2013). Rodent offspring of dams fed a HFD have impaired glucose tolerance, adipokine dysregulation, and hyperphagia, even when fostered by dams fed a normal diet immediately after birth (Desai et al. 2014). Evidence also suggests that children of obese mothers tend toward obesity (Whitaker 2004). The maternal “over‐nutrition” developmental programming hypothesis posits that maternal diet influences fetal developmental programming and outcomes in the offspring (Catalano and Ehrenberg 2006).

We hypothesized that maternal HFD would program the offspring toward a nonallergen sensitized AHR phenotype. Specifically, adult offspring of dams fed a HFD would have increased AHR compared to offspring of dams fed normal‐fat diet (NFD). Investigating our hypothesis involved a three‐step objective; first, establish a murine model of dams fed HFD and NFD previously used in AHR studies; second, cross offspring to the same diet as the dam or cross wean offspring to the opposing fat content diet, creating four cohorts of pups; and third, to evaluate AHR by cholinergic challenge in mature offspring. Additional secondary endpoints included: IL‐6 in bronchial alveolar lavage fluid (BALF), differential cell counts, total protein, and lung histology.

## Methods

### Mice and diets

**Figure 1:**
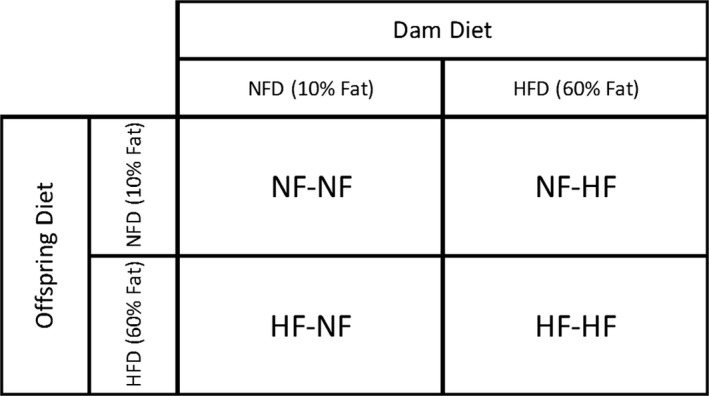
Schematic of maternal and postwean diets. Dams were fed NF or HF diet for 8 weeks prior to first breeding and remained on the diets during the course of their pregnancy and while nursing. Pups were weaned at 4 weeks of age and maintained on a HF or NF diet, creating four distinct diet cohorts. NF, normal‐fat; HF, high‐fat.

Four‐week‐old female Balb/cByJ mice were obtained from Jackson Laboratory (Bar Harbor, MA). Purified nutrient matched diets were obtained from Research Diet Incorporated (New Brunswick, NJ). To investigate a maternal diet high in saturated fats, the female mice were fed either a HFD containing 60% of Calories from lard or a NFD containing 10% of Calories from lard; these are matched purified ingredient diets specifically designed for diet induced obesity (DIO) modeling. 60% HFDs have been previously used in DIO induced AHR models (Shore 2007) this particularly high percentage fat was used as the Balb/c strain is moderately resistant to DIO (Nishikawa et al. 2007; Montgomery et al. 2013). Females were placed on their respective diets for 8 weeks prior to first breeding and remained on the diets during the course of their pregnancy and while nursing. Diets were irradiated by the manufacturer in accordance with university policy. They were purchased in small lots to avoid expiration, stored under refrigeration and changed every 4 days. Dams produced no more than three litters. To investigate HFD and NFD of offspring after weaning, on the 28th day of life, offspring were weaned to either the same diet as the dam or to the alternate diet. Thus, offspring of dams fed HFD or NFD were weaned to a NF or HFD creating four different pre and postnatal diet cohorts (Fig. 1). All experiments were performed with the approval of the OHSU Institutional Animal Care and Use Committee.

### Lung mechanics and cholinergic challenge

Mice received an intraperitoneal injection of ketamine (100 mg/kg) and xylazine (10 mg/kg). Once sedated, mice were orally intubated and ventilated using a previously described custom ventilator at a rate of 120 breaths per minute, a tidal volume range of 200–230 *μ*L, and positive end expiratory pressure of 3 cmH_2_O (Ewart et al. 1995; MacDonald et al. 2009). The mice then received an intramuscular injection of succinylcholine (1 *μ*g/g body weight) to prevent spontaneous ventilation. Inspiratory flow and pressure were measured and recorded with differential pressure pneumotachograph (part # MLT1L) and pressure transducer (part#:MLT0670) from AD Instruments (Colorado Springs, CO). The ventilator is capable of producing end‐inflation holds and the end‐inflation technique was used to measure resistance of the respiratory system (Rrs).

Cholinergic challenge was performed during mechanical ventilation with inline nebulization of methacholine (MCh) (Sigma Aldrich, St. Louis, MO). Twenty microliter of PBS, 500 mmol/L, and then 1 mol/L MCh were delivered with an Aerogen micronebulizer (part# AG‐AL1100, Kent Scientific Company, Torrington, CT). The nebulizer power was directly controlled by the expiratory solenoid circuit such that nebulization occurred only during inspiration (Macdonald et al. 2012). End‐inflation holds were performed 1 min after nebulization. Several end‐inflation holds were captured on computer hard drive using LabChart software (AD Instruments). Reproducible breaths were selected for lung mechanics analysis. At least 3 min elapsed between the doses of MCh. Mean Rrs was compared between diet cohorts following each nebulization.

### BALF collection

BALF was collected from intubated mice after the chest was opened using three 1 mL washes of cold Hank's Balanced Salt Solution (HBSS). The three washes were spun at 2000 rpm for 10 m and the supernatant was removed and stored at −80°C. Cell pellets were resuspended in HBSS with 1% fetal bovine serum and a total cell count was conducted using a hemocytometer. Cells were spun onto slides using a standard preparation method with a Shandon Cytospin 3 (Cheshire, U.K.). Slides underwent Wright‐Giemsa staining (Fisher Scientific, Kalamazoo, MI) and differential cell counts of at least five hundred cells per animal were performed. Total protein analysis was performed on BALF supernatant using a BCA Protein Assay Kit (Thermo Scientific Pierce, Waltham, MA).

### Complete blood counts

Blood samples from offspring were collected at 10 weeks of age and complete blood counts were performed with an automated Hemavet 950 system (Drew Scientific, Miami Lakes, FL).

### ELISA cytokine analysis

IL‐6 concentration of the BALF (R&D Systems, Minneapolis, MN) was measured by ELISA according to manufacturer's protocol. Only the first 1 mL wash from the BALF collection was analyzed to avoid dilution of the sample.

### Lung histology

Lung anatomy was studied by uniformly inflating the whole lung with a mixture of 4% paraformaldehyde (PFA) and 1% low melt agarose. Inflated lungs were fixed in 4% PFA overnight and then stored in 70% ethanol until paraffin block embedding. Prior to embedding, the lung was bisected in the coronal plane. Five micrometer sections were cut and mounted on slides. Sections were stained with Masson's trichrome for microscopic assessment of lung morphology by two blinded investigators. All imaging was performed on an Olympus BX51 microscope equipped with a DP71 camera (Olympus America, Center Valley, PA).

### Liver histology

Intact whole livers were removed from mice and fixed for 24 h in 4% PFA and then stored in 70% ethanol prior to being embedded in paraffin blocks. Once embedded in paraffin, 5 *μ*m sections were cut and mounted on slides. Sections were stained with hematoxylin and eosin (H&E) for assessment of liver steatosis differences between diet groups. All imaging was performed on an Olympus BX51 microscope equipped with a DP71 camera (Olympus America).

### Glucose tolerance

Multiparous dams fed their respective diets for at least 32 weeks underwent a fasting glucose tolerance test. Mice were fasted for 6 h, baseline glucose readings obtained and then they were given an IP injection of d‐glucose (2 mg/g body weight). Glucose readings were taken 15, 30, 60, and 120 min after injection. Glucose was measured using a ONETOUCH Ultra Mini glucometer (Johnson & Johnson, New Brunswick, NJ) with commercially available UniStrip1 test strips (UniStrip Technologies, Charlotte, NC).

### Whole body composition analysis

Mouse body composition analysis was performed on 4‐week‐old offspring (prior to weaning) of both HFD and NFD pregnancy using a 4‐in‐1 small animal MRI (Echo Medical Systems, Houston, TX).

### Statistical analysis

Differences in all measured outcomes between the diet cohorts were compared using one‐way analysis of variance (ANOVA) and Bonferroni post hoc analysis or *t*‐test.

## Results

### Airway hyperreactivity

**Figure 2:**
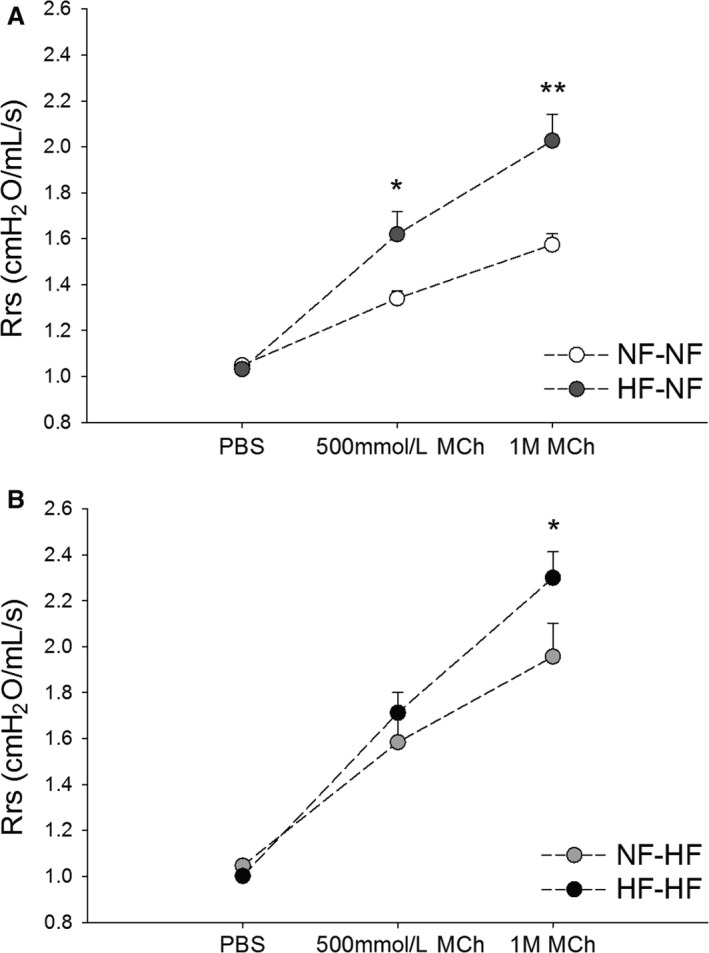
(A and B) Pre and postnatal HFDs affect response to measured resistance of the Rrs of offspring undergoing methacholine challenge. Diet cohort legend: NF is NF content diet, HF is HF content diet. The first abbreviation indicates the maternal diet, the second following the hyphen indicates the postnatal diet. (A) NF‐NF (*n* = 24) and HF‐NF (*n* = 12); (B) NF‐HF (*n* = 14) and HF‐HF (*n* = 23) offspring at 10 week of age. Rrs was measured following nebulization of PBS, 500 mmol/L and 1 mol/L MCh. *P <* *0.05, **P <* *0.01. Rrs, respiratory system; NF, normal‐fat; HF, high‐fat; MCh, methacholine.

There was no statistical difference in baseline resistance of the Rrs, following vehicle nebulization of PBS across all four diet cohorts (*P* = 0.12) at 10 weeks of age. Challenge with 500 mmol/L MCh led to a significant difference of Rrs (*P* = 0.0016) across all four diet groups. Post hoc analysis revealed that if maternal diet was HF and the offspring were then weaned to NF chow, the difference in Rrs was significant (*P* = 0.002) (Fig. 2A). Weaning NFD dam offspring to HFD also led to a significant difference (*P* = 0.0032) in Rrs. Offspring exposed to HFD during both the maternal and postnatal periods also produced significant (*P* = 0.0002) differences in Rrs compared to control diets. However, at 500 mmol/L MCh, there was no significant difference between continuous HFD and added HFD at weaning (*P* = 0.33) (Fig. 2B). During the 1 mol/L MCh challenge, Rrs remained significantly different across all four cohorts (*P* < 0.0001). In the post hoc analysis of difference in Rrs, weaning maternal HF pups to NF maintained significant difference (*P* = 0.0002) (Fig. 2A). Also, NF maternal diet weaned to HF had a significant difference in Rrs (*P* = 0.0008). HFD in both the maternal and postnatal periods produced significant change in Rrs (*P* < 0.00001) compared to control diet. At this level of MCH, the continuous HFD offspring had increased Rrs compared to maternal NF pups weaned to HFD (*P* = 0.048) (Fig. 2B). Separate analysis comparing different response to Rrs and MCh challenge by gender did not detect any significant differences (not shown).

### Unique airway reactivity in HF‐HF subset

A subgroup of HF‐HF offspring had significantly elevated baseline airway resistance. This difference would likely effect MCh response, thus we analyzed this data separately and compared it to the NF‐NF cohort (Table 1). A significant difference was seen at both the 500 mmol/L and 1 mol/L MCh dose (*P* < 0.01; *P* < 0.001, respectively).

**Table 1:** Resistance of the Rrs in 10‐week‐old mice showing response to PBS, 500 mmol/L, and 1 mol/L MCh

	PBS	500 mmol/L MCh	1 mol/L MCh
NF‐NF	1.05 ± 0.011	1.34 ± 0.033	1.57 ± 0.049
Male (*n* = 11)	1.06 ± 0.016	1.30 ± 0.026	1.51 ± 0.040
Female (*n* = 13)	1.04 ± 0.015	1.37 ± 0.057	1.64 ± 0.080
HF‐NF	1.03 ± 0.024	1.62 ± 0.099*	2.03 ± 0.140**
Male (*n* = 5)	1.047 ± 0.040	1.61 ± 0.140	2.06 ± 0.240
Female (*n* = 7)	1.021 ± 0.030	1.62 ± 0.120	2.01 ± 0.200
NF‐HF	1.05 ± 0.023	1.58 ± 0.084*	1.96 ± 0.110**
Male (*n* = 7)	1.01 ± 0.024	1.66 ± 0.150	1.94 ± 0.152
Female (*n* = 7)	1.08 ± 0.034	1.51 ± 0.082	1.97 ± 0.166
HF‐HF	1.00 ± 0.015*	1.71 ± 0.088***	2.30 ± 0.11***
Male (*n* = 10)	0.99 ± 0.020	1.82 ± 0.150	2.43 ± 0.186
Female (*n* = 13)	1.01 ± 0.021	1.63 ± 0.104	2.20 ± 0.141
HF‐HF	1.39 ± 0.025***	2.73 ± 0.30**	3.59 ± 0.36***
Male (*n* = 5)	1.40 ± 0.034	2.34 ± 0.500	3.49 ± 0.545
Female *(n* = 4)	1.40 ± 0.040	3.03 ± 0.244	3.67 ± 0.548

The shaded row represents the cohort of HF‐HF that had significantly higher baseline Rrs compared to the control cohort. Subanalysis between genders revealed no differences. Values shown are mean Rrs (cmH_2_O/mL per second) ± SEM. Rrs, respiratory system; MCh, methacholine; NF, normal‐fat; HF, high‐fat.

*P <* *0.05, **P <* *0.01 and ***P <* *0.001 compared to NF‐NF.

### Inflammatory cells, total protein, and IL‐6 in the lung

**Figure 3:**
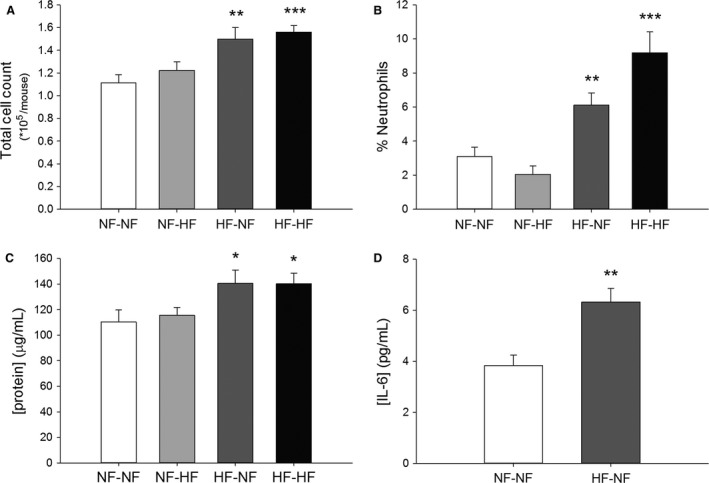
(A–D) BALF collected from maternal HFD diet 10‐week‐old offspring induces change in total cell count, neutrophil percentage, total protein, and IL‐6 concentration. Diet cohort legend: NF is NF content diet, HF is HF content diet. The first abbreviation indicates the maternal diet, the second following the hyphen indicates the postnatal diet. (A) Total cell count of NF‐NF (*n* = 14), NF‐HF (*n* = 12), HF‐NF (*n* = 17), and HF‐HF (*n* = 12). (B) Percentage neutrophils of NF‐NF (*n* = 8), NF‐HF (*n* = 7), HF‐NF (*n* = 10), and HF‐HF (*n* = 10). (C) Total protein content of NF‐NF (*n* = 8), NF‐HF (*n* = 7), HF‐NF (*n* = 6), and HF‐HF (*n* = 8). (D) IL‐6 concentration of NF‐NF (*n* = 4) and HF‐NF (*n* = 4). All bars indicate mean ± SEM. *P <* *0.05, **P <* *0.01 and ***P <* *0.001. BALF, bronchial alveolar lavage fluid; NF, normal‐fat; HF, high‐fat.

At 10 weeks of age, total cell counts from BALF revealed increased cell infiltrate in the offspring of HFD dams weaned to both HFD and NFD (Fig. 3A). The offspring of HFD dams had increased neutrophil percentage compared to NF‐NF offspring (Fig. 3B). Differential cell counts did not reveal a difference in eosinophil populations among diet cohorts. BALF total protein was significantly increased among the offspring of HFD dams weaned to both HFD and NFD compared to offspring of NFD dams weaned to NFD (Fig. 3C). Cytokine analysis of BALF revealed a significant difference in IL‐6 concentration between the NF‐NF and HF‐NF cohort (Fig. 3D). We were unable to complete the other cohort analysis for IL‐6. Complete blood counts obtained from offspring at 10 weeks of age revealed no significant differences in RBC, WBC, or differential counts between groups (not shown).

### Lung and liver histology

**Figure 4:**
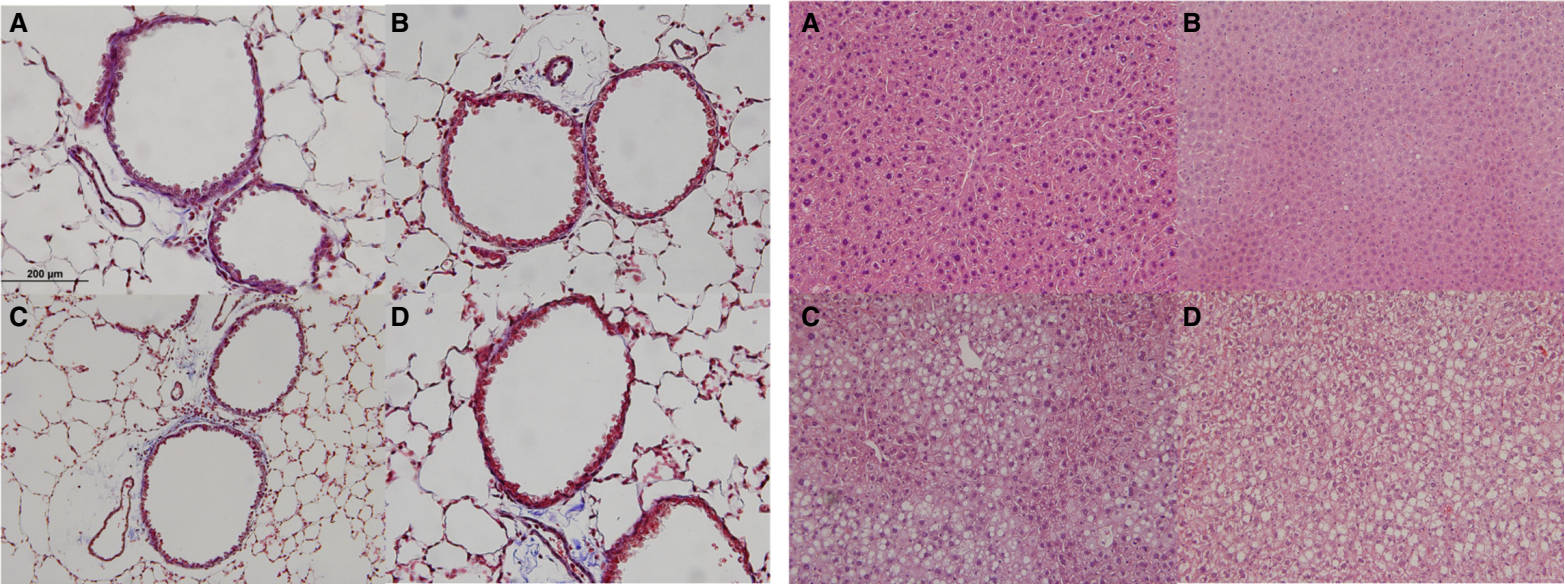
(A) Lung sections from diet cohorts do not show overt inflammation or gross structural changes. Representative images of Masson's trichrome stained lungs (40X). (B) Liver sections stained hematoxylin and eosin stained liver (20X) reveal vacuolization only in offspring cohorts actively consuming HFD. All images are of offspring at 10 weeks of age. Diet cohort legend: NF is normal fat content diet, HF is high fat content diet. The first abbreviation indicates the maternal diet, the second following the hyphen indicates the postnatal diet. A. NF‐NF, B. HF‐NF, C. NF‐HF and D. HF‐HF.

Masson's trichrome stained lung sections of 10‐week‐old offspring demonstrated no gross differences in lung structure, airway thickness, or presence of inflammatory cells (Fig. 4A). Evidence of liver steatosis was only seen in the offspring weaned to the HFD. H&E stained sections of livers showed evidence of vacuolization in offspring weaned to HFD (Fig. 4B).

### Offspring weight and fat mass by diet

**Figure 5:**
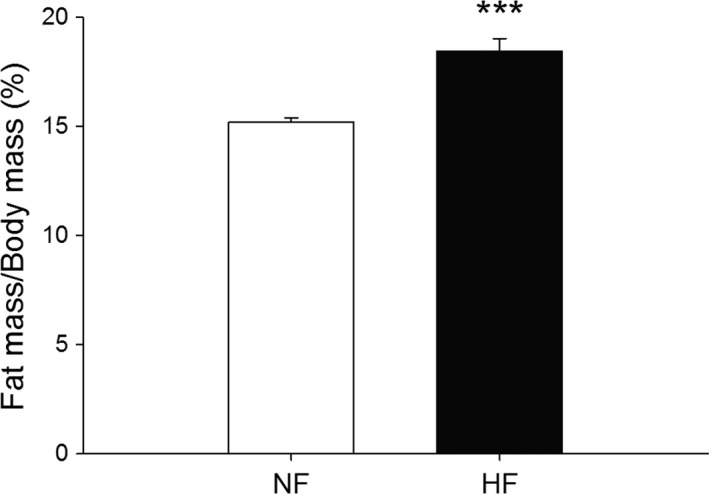
Fat mass to body weight ratio of 4‐week‐old offspring from HFD dams (*n* = 20) is greater than NFD dams at weaning (*n* = 20). ***P =* *0.001.

Offspring from HFD dams were observed at birth and noted to be born at the same gestational age and were visually identical to pups from NF dams. Pups were not weighed or handled prior to weaning to avoid rejection by dams. Offspring of HFD dams were 13.5% larger (*P* < 0.001) at 4 weeks than offspring of NFD dams. At 10 weeks of age, their weights were no longer significantly different when grouped by postwean diet (Table 2). Fat mass to body weight ratio of 4‐week‐old offspring of NF and HFD dams showed increased adiposity in the offspring of HFD dams (*P* < 0.001) (Fig. 5) prior to weaning.

**Table 2:** Mouse weights by diet cohort and gender at 10 weeks

	10‐week‐old weight (g)	*N*
NF‐NF		
Male	25.18 ± 0.44	18
Female	20.82 ± 0.40^a^	20
NF‐HF		
Male	28.39 ± 0.77	12
Female	23.01 ± 0.69^a^	15
HF‐NF		
Male	26.61 ± 0.59	14
Female	20.96 ± 0.47^a^	16
HF‐HF		
Male	27.76 ± 0.72	17
Female	21.13 ± 0.39^a^	18

Values are mean weights ± SEM. NF, normal‐fat; HF, high‐fat.

a *P *<* *0.001.

### Dam weight and glucose tolerance testing

**Figure 6:**
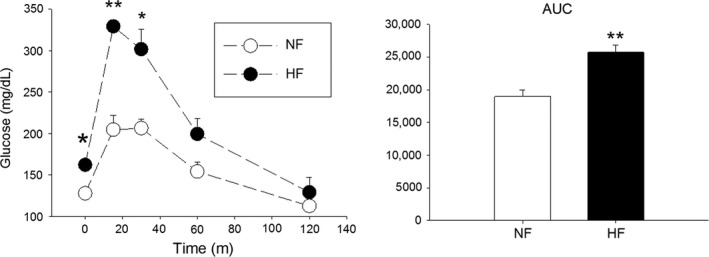
Glucose tolerance test in nonpregnant, multiparous dams fed HF diet (*n* = 3) reveals impaired glucose tolerance compared to NF (*n* = 4) dams. Glucose concentrations were taken after a 6 h fast, and 15, 30, 60 and 120 min following an IP injection of glucose. The AUC of the two groups was also calculated.*P <* *0.05, and **P <* *0.01. HF, high‐fat; NF, normal‐fat; AUC, area under the curve.

After 70 days on diet the mean weight of the dams on HFD (*n* = 4) was 22.2 g (SEM ±0.73), on the NFD (*n* = 4) the mean weight was 22.5 g, (SEM ±0.46), the difference was not significant (*P* = 0.37). Multiparous dams that had been on HFD for at least 32 weeks had elevated glucose levels 15 and 30 min after IP glucose injection. This altered glucose metabolism is also seen in area under the curve calculations (Fig. 6). We also examined glucose tolerance testing in 10‐week‐old offspring, as expected both cohorts that continued HFD had impaired glucose tolerance but both cohorts on NFD were normal (not shown).

## Discussion

The principal finding of this study is that offspring from a murine model of maternal HFD prior to and during pregnancy and nursing weaned to NFD at 4 weeks of age show increased AHR by cholinergic challenge at 10 weeks of age compared to offspring of dams fed NFD. AHR is strongly associated with asthma and responses to inhaled stimulants such as MCh are correlated with asthma severity (National Heart Lung, and Blood Institute 2007). Thus, our finding demonstrates a direct causal relationship between maternal HFD and the clinically relevant finding of AHR in adult offspring consuming a NFD. This supports observational studies associating maternal obesity and early life wheezing and asthma risk in children if a diet high in saturated fats significantly contributes to maternal obesity (Macdonald et al. 2013; Pike et al. 2013; Forno et al. 2014; MacDonald et al. 2016).

In our model of maternal HFD exposure, Balb/cByJ offspring are exposed to a HF maternal diet for 21 days in utero and via nursing or per os until the 4th week of life (28 days) before being weaned to NF or a continued HFD. Weaning was completed at the fourth week of life to ensure the pups could consume the diet without need for softening the chow (Silver 1995). Pup consumption of the HFD prior to weaning was likely limited as offspring nurse through the third week and onto the fourth (Silver 1995). Variation in the proportions of fatty acids has been found in expressed milk from mice on HFD and NFD, but total fat content is similar (Silverman et al. 1992). In a rodent model of maternal HFD pregnancy, metabolic and cardiovascular consequences were shown to be a result of HFD in utero and during lactation (Desai et al. 2014). Importantly, HFD exposure during pregnancy alone also produced similar phenotypic effects, suggesting in utero HFD exposure is sufficient to alter offspring phenotype, but that continued diet exposure after parturition further effects offspring health. We reasoned that the most relevant choice in translating our initial model to human health was to continue HFD in the nursing dam and to institute diet modification at weaning to study effects of offspring diet on AHR. Future models will study the effect of cross‐fostering offspring.

A second key finding is the observation that adult offspring of HFD pregnancy that remained on HFD after weaning had a greater response to cholinergic challenge at 10 weeks of age compared to offspring of NFD dams fed a HFD postweaning at the 1 mol/L MCh dose. This is an important postnatal diet comparison as HFD in newly weaned adult rodents is a known contributor to AHR (Shore 2007). We now show increased AHR in offspring of both early and combined in utero and later life HFD exposure. By varying diets at weaning, our results differ from a previously published rat model of HFD exposure at pregnancy onset showing increased airway reactivity in weanling offspring and adult rats that continued on HFD (Griffiths et al. 2016). Those authors reported but did not show any differences between HFD cross at weaning in the offspring. Our results show increased AHR in HFD offspring that continued on HFD compared to offspring fed HFD at weaning. Our results are comparable to a recent model of murine pre and postnatal hyperalimentation showing increased AHR without alteration in bronchial structure that may be due in part increased T_h_17 cells (Dinger et al. 2016).

DIO in the adult C57/BL6 mouse has been shown to produce increased AHR as measured by the change in resistance of the lung (*R*_L_) (Shore 2007). Changes in AHR phenotype in C57/BL6 DIO models are not typically seen until the body mass had increased by ~45%, which occurred after 30 weeks of HFD consumption (Shore 2007). Thus, body mass increases and duration of diet are important in adult C57/BL6 mouse DIO AHR models. In our studies, the offspring of HFD pregnancy were 13.5% (*P* < 0.001) larger than those of NFD pregnancy at 4 weeks. However, at 10 weeks of age, the HFD litters weaned to NFD were not significantly larger than NFD litters weaned to NFD; nor were HFD litters weaned to HFD significantly larger than NFD litters weaned to HFD (Table 2). Meta‐analysis of human data found that low gestational age, rapid infant weight gain and low birth weight were most associated with school age asthma (Sonnenschein‐van der Voort et al. 2012, 2014). Our data demonstrate that early life weight gain and birth weight and not necessary body weight at the time of testing are associated with AHR. Body mass is a poor indicator of fat mass. To further understand the role of in utero and early‐life HFD exposure in our model, we also examined the fat mass/body weight ratio by whole body composition analysis at the age of weaning. At 28 days of age, offspring of dams fed HFD had a greater ratio of fat mass to body weight mass compared to offspring of dams fed NFD. However, at 10 weeks of age we did not detect any differences in the fat mass to body weight ratio. HFD consumption by rodents is also associated with liver steatosis. In our model, only diet cohorts weaned to HFD had gross evidence of increased hepatic fat deposition at 10 weeks of age.

In some adult mouse models of obesity and AHR, the thorax is opened to remove mechanical contribution from AHR testing. Proposed mechanical causes for asthma symptoms in obese humans including increased chest mass resulting in higher respiratory rates, lower tidal breathing, and reduced functional residual capacity (Shore 2008). In our model, offspring did not have significantly different body masses between cohorts at the time of testing we elected to leave the thorax closed during lung mechanics testing.

The HF‐HF cohort with elevated baseline Rrs underwent separate analysis as this could have been a confounder in the analysis. Our previous experience with a C57BL/6 HFD pregnancy model produced a similar elevation of baseline resistance in the offspring of HFD pregnancy but we were unable to determine the cause (Macdonald et al. 2013). Further investigation of this subtype will be needed.

To address the aspect of our hypothesis that unintentional allergen sensitization did not contribute to AHR, we examined BALF, serum, and airway histology from offspring of dams fed NF and HFDs. This is important as early onset childhood asthma is most often associated with allergic sensitization and mouse models of allergen sensitized AHR are well characterized (Nials and Uddin 2008). Our model showed a complete absence of stigmata of airway inflammation typically seen in allergen sensitized mouse models of asthma. Allergen sensitization induces AHR and lung inflammation resulting in airway hyperplasia and eosinophilia (Boyce and Austen 2005). These findings were not present in our cohorts either by lung histology (Fig. 3) or complete blood counts (data not shown). In this study, none of the mice underwent allergen sensitization. The animals were maintained in air‐filtered housing and had no changes in environmental conditions other than weekly bedding and the diets as noted. There were no infectious disease outbreaks in any colony during the study period. All mice regardless of diet thrived and maintained expected growth during the study periods. Sentinel animals did not reveal evidence of pathogens or infection in the housing unit.

The increased total cell count in the BALF of all offspring from dams fed HFD is a novel finding. In our study, all offspring of dams fed HFD had higher BALF total cell counts compared to NF pregnancy diet. Importantly, this persisted in the HF pregnancy offspring weaned to the NFD (Fig. 3), suggesting an in utero programming effect of HFD exposure. Previous reports of adult DIO in C57BL/6J mice (Shore 2007) did not show a significant change in BALF total cell counts, whereas a DIO model using a lower fat diet (45%) in the same mouse strain revealed increases in both total cell count and neutrophilic infiltration (Jung et al. 2013). Further classification of BALF cells, particularly macrophage subtype in the offspring of HFD represents a clear target for future investigation.

Another important observation in this study is the increased percentage of neutrophils in the BALF of offspring of dams on HFD (Fig. 2B). Neutrophils in the airway have been reported in both obese asthma and steroid‐resistant asthma (Haldar et al. 2008; Telenga et al. 2012). However, the presence of significant countable neutrophils in BALF is not common in most control conditions of mice. We did open the chest prior to BALF washing and this has been reported to significantly increase neutrophil recruitment in BALF of mice (Lamb and Evans 2011). Additionally, we performed three large volume of wash. To ensure consistency the counts were performed by the same person and in no particular order. The distribution of cell counts was consistent and reflects our findings.

The neutrophil findings may be related to our observation of an increase in IL‐6 in the BALF (Fig. 2D) of the offspring of HFD weaned to NFD. IL‐6 has been studied in obesity and circulating levels are correlated with levels of adiposity (Cottam et al. 2004). Secretion of IL‐6 is linked to both adipocytes and macrophages although the mechanism for increased circulating levels in obesity is not fully understood (Fantuzzi 2005). Unfortunately, we were unable to examine other inflammatory cytokines in BALF. In our model, offspring of dams fed HFD also exhibited higher total protein concentration in their BALF than the normal diet cohort. Total protein in BALF has been used previously to demonstrate alveolar capillary leak or airway inflammation following infection (Kumar et al. 2014), although it is not elevated in human atopic studies (Schock et al. 2003). Collectively the BALF findings correlate well to a limited human investigation of nonallergic asthma where MCh reactive subjects had increased cell counts, increased neutrophils, increased total proteins, and IL‐6 expression in their BALF (Mattoli et al. 1991). Unfortunately the body mass and dietary history of those human subjects was not reported.

A strength and limitation of our model is the observation that the dams did not gain significant body mass on the HFD and thus do not have increased body habitus or significant weight differences when compared to dams on NFD (not shown). The Balb/C mouse strain is known to have an “intermediate” DIO phenotype, such that metabolic consequences of HFD occur without significant gain in body habitus as is seen in other strains (Nishikawa et al. 2007; Montgomery et al. 2013). This was a deliberate choice as our previous experience suggested that offspring of C57BL/6 dams fed HFD suffered from premature birth, had smaller birthweights, required cross fostering during nursing, and had craniofacial defects (Macdonald et al. 2013). In those pilot experiments we noted abnormal baseline Rrs and alveolar development. A recent publication expands and details these types of findings (Mayor et al. 2015).

Importantly, changes in body mass alone do not reflect the adiposity of a given animal on a particular diet. Balb/C mice fed a HFD have increased adipose density by DEXA scan, increased fat pad mass, increased liver weight, and enhanced markers of adipose tissue inflammation that was similar to other mouse strains which gain significant body mass (Nishikawa et al. 2007; Montgomery et al. 2013). Our data reveal glucose intolerance (Fig. 6) and evidence of liver steatosis in the dams fed HFD, which suggests that this Balb/cByJ mouse model preserves some the metabolic stigmata of obese pregnancy. Unlike human pregnancy, no effort was made to control glucose with insulin, and the dams on HFD demonstrated impaired glucose tolerance. These findings suggest the dietary intake of saturated fats during pregnancy and their metabolic consequences, and not solely the maternal body habitus, are contributing to the offspring AHR phenotype. Notably, the Balb/cByJ dams fed HFD produced litters of normal appearance, similar birth size and term gestation pups. This observation parallels a large human birth cohort study that found most obese pregnancies carry to term and produce normal to macrosomic offspring (Sebire et al. 2001).

In conclusion, our results demonstrate that maternal HFD programs increased AHR in the offspring. As hypothesized, the offspring of dams fed a diet high in saturated fats had increased AHR compared to offspring of dams fed NFD at 10 weeks of age. Significant increases were seen in BALF total cell infiltrate, BALF neutrophil count, BALF total protein, and IL‐6 in the BALF of offspring of HFD dams.

## Conflict of Interest

None declared.
